# Perceptions of HIV transmission and pre-exposure prophylaxis among health care workers and community members in Rwanda

**DOI:** 10.1371/journal.pone.0207650

**Published:** 2018-11-26

**Authors:** Immaculate Kambutse, Grace Igiraneza, Onyema Ogbuagu

**Affiliations:** 1 Department of Medicine, University Teaching Hospital of Kigali, Kigali, Rwanda; 2 Yale AIDS Program, Section of Infectious Diseases, Department of Medicine, Yale University, New Haven, Connecticut, United States of America; Thammasat University Hospital, THAILAND

## Abstract

There are too many new HIV infections globally with 1.8 million persons infected in 2016 alone. Pre-exposure prophylaxis (PrEP) holds potential to decrease new infections and is synergistic with efforts currently in place to achieve an end to the AIDS epidemic in Sub-Saharan African, but uptake is limited. Given its novelty, assessing the beliefs and attitudes of healthcare professionals and members of the community towards HIV transmission and PrEP will be helpful to inform implementation efforts. Study was a random survey of 201 community members and 51 healthcare providers, carried out at multiple community sites in Huye district, Southern Province, Rwanda and at Kigali University Teaching Hospital (KUTH). The study findings are that there are still misconceptions about HIV in the community with some respondents believing that HIV is due to punishment from God (5.4%), poverty (3.0%), smoking cigarettes (1.0%), drinking alcohol (2.0%), punishment from ancestors (1.0%) and witchcraft (1.5%), and that its transmission is by mosquito bites (10.9%), sharing food or drinks with a HIV infected person (6.5%) or as a result of carelessness (47.8%). More than 50% of respondents from both groups had insufficient knowledge regarding PrEP, but expressed some interest in PrEP (82.6% of the respondents from the community and 86.5% of the health workers). However, some healthcare workers felt that promotion of safe sex practices (74.5%), HIV testing and treating HIV infected patients (60.8%) would work better than PrEP to decrease new HIV infections. Barriers to PrEP implementation included perceived stigma, delayed access to prevention services at the health facilities while personal-level concerns included lack of family support, reluctance to take a medication daily and fear of being perceived as having HIV. This study showed that health care workers and community members are willing to utilize PrEP in Rwanda, but many challenges exist including limited knowledge about PrEP, stigma, provider and system level service delivery barriers at health facilities among others. More studies are needed to assess ways of addressing and /or eliminating these barriers.

## Introduction

In 2016, there were 1.8 million new HIV infections and 1 million deaths related to AIDS[1]. Rwanda, has an estimated HIV prevalence of 3% among the adult general population, with a disproportionately higher prevalence in urban areas such as Kigali (7%.) and among certain risk groups such as sex workers (51%)[2]. The primary driver of HIV infection in Rwanda is heterosexual transmission, with no data on the prevalence of HIV among men who have sex with men (MSM).

Through public health efforts, Rwanda has been able to keep its HIV incidence low, but there is a long way to go to achieve zero new infections. To achieve this ambitious target, Rwanda has set national strategic plan with the goal to reduce new HIV infections by two thirds by June 2018 (from 6000 to 2000 cases per year), cut HIV related deaths in half from 5000 to 2500 during the same time frame; and reduce HIV related morbidity.[3] In Rwanda, HIV prevention takes multiple forms but is not currently inclusive of HIV PrEP. Male circumcision (168,980 males as of 2016), prevention of mother to child transmission (PMTCT) program scale up (97.7% coverage among its health facilities in 2016), and population education where condom promotion, abstinence and monogamy are encouraged are the primary modes of HIV prevention employed.[4] In the same vein, the vast majority of HIV positive Rwandans who are diagnosed are on antiretroviral therapy regardless of their CD4 counts (implemented since July 2016) including those in serodifferent relationships, consistent with WHO guidelines, such that treatment as prevention is robust.[5] Prevention programs have also been instituted for high risk groups including female sex workers (FSWs).[5] However, stigma around sexual minorities and other high risk groups persist and limit the reach of these prevention approaches and significant numbers of new HIV infections continue to occur.

**As** current strategies that have been employed to prevent HIV transmission have not resulted in optimal reduction in incident cases, there is, therefore, an urgent need for novel innovative and effective preventive strategies such as pre-exposure prophylaxis (PrEP), which involves the utilization of antiretroviral medications (ARVs) by at-risk persons to prevent HIV acquisition [6].

Multiple studies conducted in sub-Saharan Africa including among sero-different heterosexual couples carried out in Uganda and Kenya (East Africa)[7, 8] and Botswana (Southern Africa),[9] showed high efficacy of PrEP in preventing HIV transmission—75% (oral tenofovir disoproxil fumarate [TDF] / emtricitabine [FTC]) and 62% (oral TDF) in the intervention groups compared to the control groups respectively. In these trials, medication adherence emerged as the key factor determining the efficacy of PrEP as suggested by trials among heterosexual females (FEM-PrEP and the VOICE trials) where lack of efficacy was attributed to low rates of PrEP medication adherence.[10, 11]

Multiple barriers exist among healthcare workers and community members that may impact the adoption and utilization of PrEP and these vary by geographical region due to cultural, religious and socioeconomic variables among others. Negative perceptions in the community about the use of PrEP are a key barrier to its roll-out including that its use is associated with HIV infection or being at high risk for disease acquisition, both of which are stigmatizing.[6] Also, certain high-risk individuals do not perceive themselves to be at risk for HIV and therefore do not consider themselves as potential users of PrEP. There is also concern among the medical community about drug costs, adverse effects, the emergence of drug resistance and sexual risk compensation behavior by users of PrEP [12, 13].

PrEP use as a HIV prevention strategy among at-risk populations, is emerging in neighboring countries including Uganda, Tanzania and Kenya. Until recently, HIV PrEP was not being offered in Rwanda and has only begun to be discussed as a prevention option, and is yet to be included in clinical guidelines. Furthermore, there is lack of data on the extent of awareness about PrEP and willingness of healthcare providers and community members to either prescribe or utilize the service respectively. Prior to implementing PrEP, it is important to identify barriers to its uptake among these stakeholders. In this study, the first of its kind in Rwanda, we evaluated the beliefs and attitudes about PrEP among health care workers and non-healthcare worker individuals in the community in order to evaluate potential barriers to its roll out and to inform where efforts should be focused to overcome them.

## Methods

This was a cross sectional study based on a structured survey of a convenience sample of randomly selected individuals, carried out at Kigali University Teaching Hospital (KUTH) in Kigali City (an urban setting) and at multiple community sites in Huye district (a semi-urban setting). Huye district, a college town located in the Southern Province of Rwanda, by virtue of its demographics, has a broad representation of reproductive age individuals in the country. Consenting participants from the community responded to researcher-administered questionnaires whereas healthcare workers’ self-administered their own questionnaires in their preferred language (English or Kinyarwanda). The questionnaires were in print form, respondents filled out questionnaires anonymously and completed ones were given to study investigators. Surveys were administered between October 2016 and January 2017.

### Study sites / population

Kigali University Teaching Hospital (KUTH) is a 390 bed, publicly funded tertiary-level teaching hospital located in Nyarugenge District in Kigali city, Rwanda whereas Huye District, located in the Southern Province, is 133 Kilometers drive South West from the capital city, Kigali. Health care workers surveyed for the study included attending physicians, resident doctors, medical officers, registered nurses and staff of voluntary counseling and testing (VCT) program working at KUTH. Community-based participants were recruited from any of the following congregate areas: markets, taxi parks, people visiting patients in the hospital, and at college campuses.

### Eligibility criteria

Respondents had to be adults (age 18 and older) and able to provide informed consent. In Rwanda, PrEP is yet to be implemented such that no respondents were PrEP users or prescribers. People surveyed as community members had to not have a medical background defined as not having ever worked or currently working in a healthcare facility of any kind in any capacity or role.

### Data collection methods and tools

The survey tools were adapted from studies conducted in similar settings [14–16]. Only one investigator conducted the survey for each group so there was no need for calibrating inter-interviewer data. The surveys were translated from English into Kinyarwanda with 2 investigators reviewing language for descriptive accuracy and were administered in either language as preferred by respondents (for both healthcare workers and community members).

For health care workers, the interviewer recruited the participants from their respective departments in KUTH during week days over a period of 4 weeks. This was carried out in Internal medicine department, including the HIV clinic (TRAC) and the department of Gynecology & Obstetrics. Recruits included Attending Physicians, Resident Doctors, and Medical Officers, Registered Nurses and voluntary counseling and testing (VCT) workers. Recruited participants were approached during morning report meetings and their respective work units such as wards and offices. The participants self-administered the questionnaires. The questionnaires were structured to assess both knowledge and attitudes of respondents about HIV transmission and prevention modalities—particularly PrEP. Questionnaires given to health care workers had questions grouped into 5 sections; demographics, knowledge and perceptions of HIV, preexisting knowledge of PrEP, attitudes towards PrEP and interest in providing PrEP (survey is attached as a supplement.)

For community members, a study investigator, trained by the lead investigator (first author), recruited participants from the market place, taxi parks, and at college campuses in Huye district over a period of one and a half months. The surveyor approached participants in the congregate areas described above, and after obtaining consent, completed the questionnaires based on participants’ responses to allow for uniformity of data collection. The questionnaire was divided into 6 sections: questions about respondent demographics, their knowledge and perceptions of cause and transmission of HIV, pre-existing knowledge of PrEP, self-interest in PrEP and perceived benefits and barriers to PrEP (survey is also attached as a supplement.)

Both groups of participants were initially provided a brief description of PrEP prior to completing questionnaires using the following terms (Table 1):

**Table 1:** Description of HIV Pre-exposure prophylaxis provided to survey respondents.

Group	Opening statement
Health care workers	*“There is a medication that is used as Pre-exposure prophylaxis (PrEP) that if taken correctly every day*, *would reduce an individual’s risk of getting HIV*. *PrEP is taken daily to prevent HIV infection*. *If taken correctly every day*, *their risk of acquiring HIV is decreased*. *The next questions ask how you feel about the idea of patients taking medicine to prevent HIV”*
Community members	“*There is a medication that is used as Pre-exposure prophylaxis (PrEP) that if taken correctly every day*, *will reduce your risk of getting HIV*. *If you took it correctly every day*, *you would be protected from getting sick with HIV for as long as you took the medication*. *You would have to go to the clinic monthly to get PrEP and have a check-up*. *The next questions ask how you feel about the idea of taking medication to prevent HIV”*

Confidentiality was maintained for both community members and health care workers, as identifying information was not collected. The survey tools utilized are included as a supplement.

### Sample size

Due to limited time and resources, a convenience sample of 252 individuals was targeted with the aim of surveying at least 200 community members and 50 healthcare workers. Everyone meeting study eligibility criteria were approached by study investigators, consenting participants were administered the questionnaire, and enrollment discontinued once targeted sample size was reached.

### Statistical analysis

Data collected from the questionnaires was entered into an Epidata database (Version 3.1) and later exported to stata 13 for statistical analysis. Patient demographics and study responses were reported as simple frequencies of total respondents and responses respectively. Specifically, correct and incorrect responses to questions with multiple-choice answers were reported as simple frequencies as well. In some cases, the multiple choice answers to survey questions allowed for selection of more than one response. Differences in the frequencies of selected questionnaire responses between healthcare workers and community respondents were assessed using Chi square test or Fisher’s exact test as indicated with a P value <0.05 considered significant.

### Ethical considerations

Authorization to conduct the study was obtained from the College of Medicine and Health Sciences Research committee (IRB) of University of Rwanda and from KUTH research Committee. Informed consent was obtained from each participant in the participants’ language of preference (English or Kinyarwanda) after adequate explanation about the aims of the study. Participants’ confidentiality was maintained and they were informed of the right to withdraw from the study at any moment upon their request. Participation was voluntary and no incentives were offered.

## Results

### Participants’ socio-demographic characteristics

Overall, 300 and 80 community members and health care workers respectively were approached by study investigators with 201 community members and 51 healthcare workers accepting to participate in the survey. Of the community members, majority of the respondents were female (53.7%). Half of the respondents were single while 42.8% were married. The mean age of the respondents was 30 years. Most of the respondents had attended at least primary school (87.1%), however, the majority were unemployed (76. 2%) [Table 2].

**Table 2:** Socio-demographic characteristics of survey respondents (N = 201).

Characteristics	Number (Frequency in %)
**Gender**	
Male Female	93(46.27%) 108(53.73%)
**Marital status**	
Married Single Partner Widowed Other	86(42.79%) 101(50.25%) 5 (2.49%) 7(3.48%) 2(1.00%)
Yes No	48(23.88%) 153(76.12%)
Ever attended school	
Yes No	175(87.06%) 26(12.94%)
Age (Mean = 29.995	
18–30 31–40 41–50 51–60	125(62.19%) 46(22.89%) 22(10.95%) 4(3.98%)
**Insurance provider**	
Community based health insurance Rwanda social security board scheme Private health insurance	185(92.04%) 9(4.48%) 7(3.48%)
Less than 30mins Less than 1 hour Less than 2 hours	136(67.66%) 62(30.85%) 3(1.49%)
On foot Taxi Personal vehicle Other	161(80.10%) 35(17.41%) 0 5(2.49%)

### Knowledge and perceptions on HIV transmission

#### Cause and transmission of HIV

**Fig 1:**
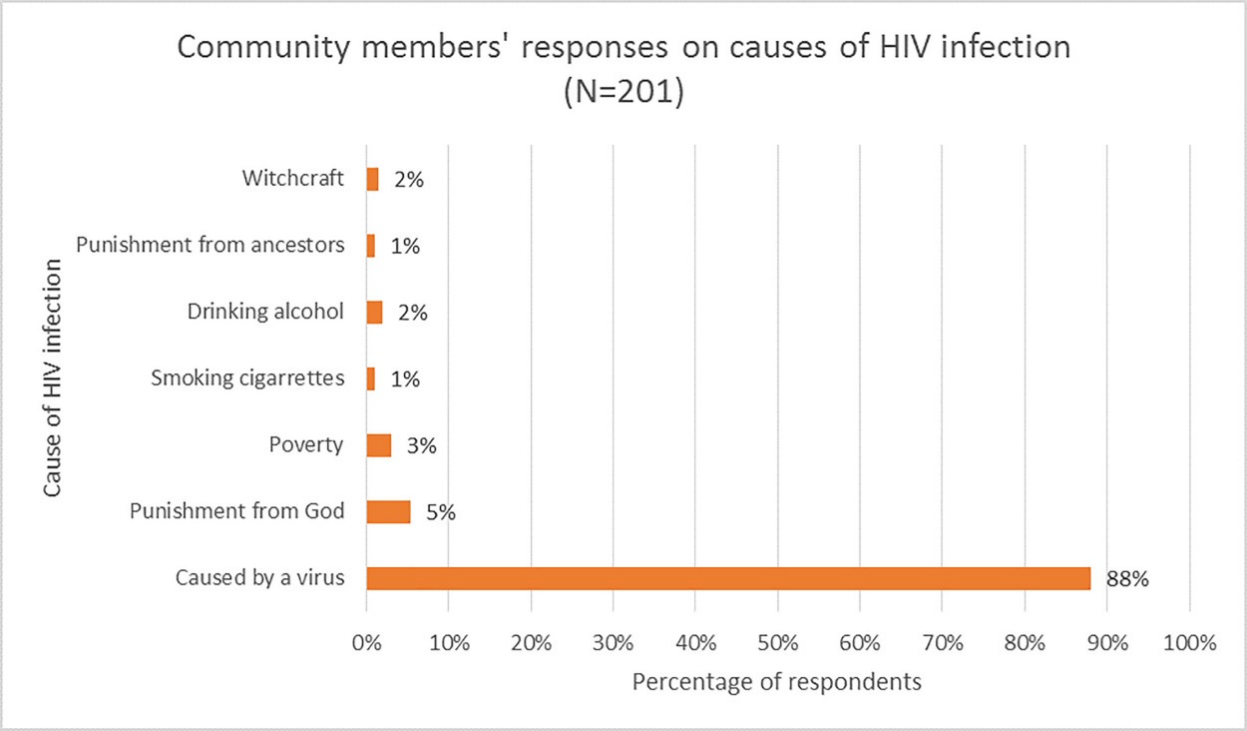
Community members’ responses on causes of HIV infection.

**Fig 2:**
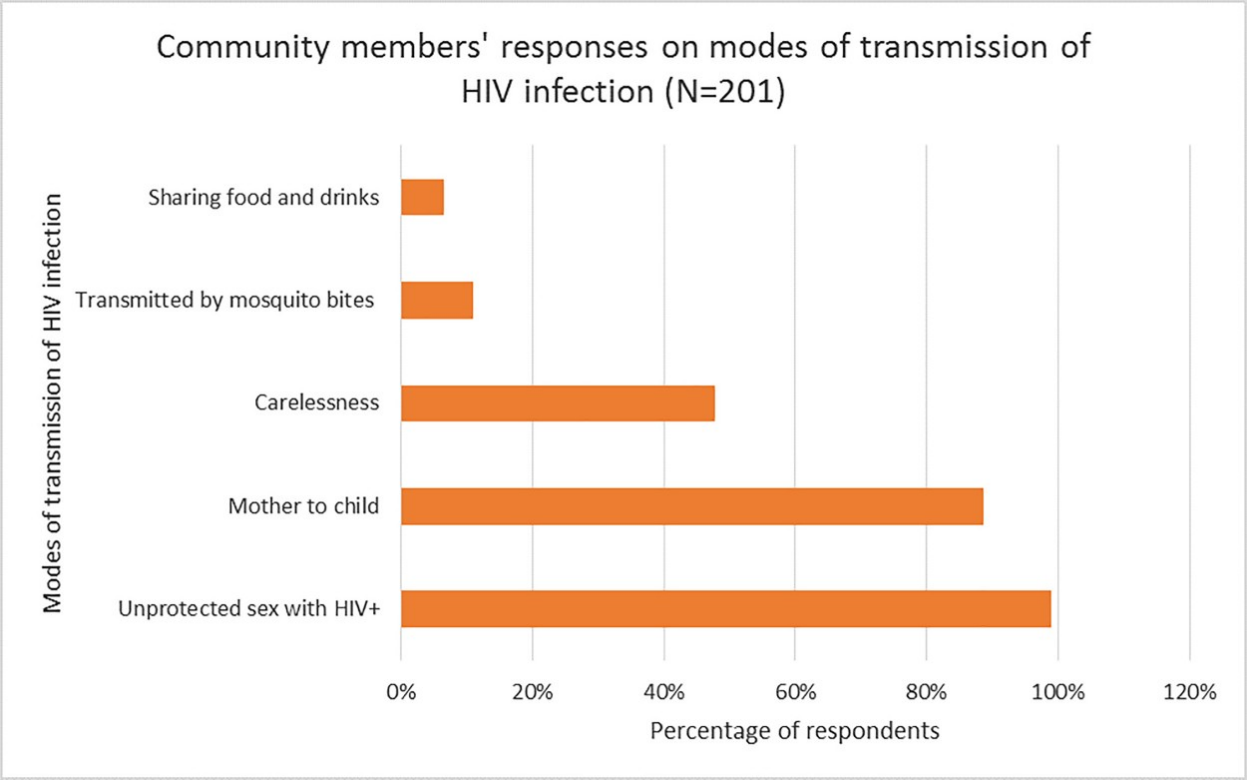
Community members’ responses on modes of transmission of HIV infection.

Overall, majority (88.1%) of the respondents from the community knew that a virus causes HIV infection, and a majority (99.1%) of the respondents had accurate knowledge about modes of transmission of HIV including unprotected sex with a person who has HIV (99.0%) and through mother to child transmission (88.6%). However, some respondents also had misconceptions about the cause of HIV disease and these included punishment from God (5.4%), poverty (3.0%), smoking cigarettes (1.0%), drinking alcohol (2.0%), punishment from ancestors (1.0%) and witchcraft (1.5%). Some respondents stated that HIV can be transmitted by mosquito bites (10.9%) and sharing food or drinks with someone who has HIV infection (6.5%). Almost half (47.8%) reported feeling that people with HIV became infected by their own carelessness [Figs 1 and 2.]

### Level of awareness about PrEP

The level of knowledge of PrEP was analyzed among all 252 respondents. Overall, more health workers (86.4%) had heard about PrEP than individuals in the community (61.7%), P = 0.0001. Pre-existing knowledge of PrEP was assessed using these questions: *“Have you ever heard about pre-exposure prophylaxis*?*”*, *“How much would you say you know about PrEP*?*”* More than 50% of respondents from both groups had insufficient knowledge regarding PrEP. This was measured by questions about PrEP offering complete protection against HIV, dosing frequency of PrEP or if PrEP can be used as a substitute for usual prevention methods like condom use.

### Interest in PrEP

Overall, majority (82.6%) of the respondents from the community expressed interest in PrEP generically when they were asked hypothetically about using PrEP if they were at risk for HIV. If PrEP use was advised by a health care worker in order to decrease one’s risk of getting HIV, an even higher percentage (93.5%) showed interested in PrEP. More males were interested in taking PrEP than females (P = 0.001). More respondents (91.0%) who have ever attended school were interested in PrEP than those who hadn’t (9. 0%) (P < 0.0001). 86.5% of the health workers expressed at least some interest in promoting PrEP. 74.5% of healthcare workers however, felt that promoting safe sex practices will be more effective than PrEP to decrease risk of HIV infections while more than half (60.8%) believed HIV testing and treating HIV infected patients is more important than offering PrEP.

### Preferred formulation and frequency of PrEP

**Fig 3:**
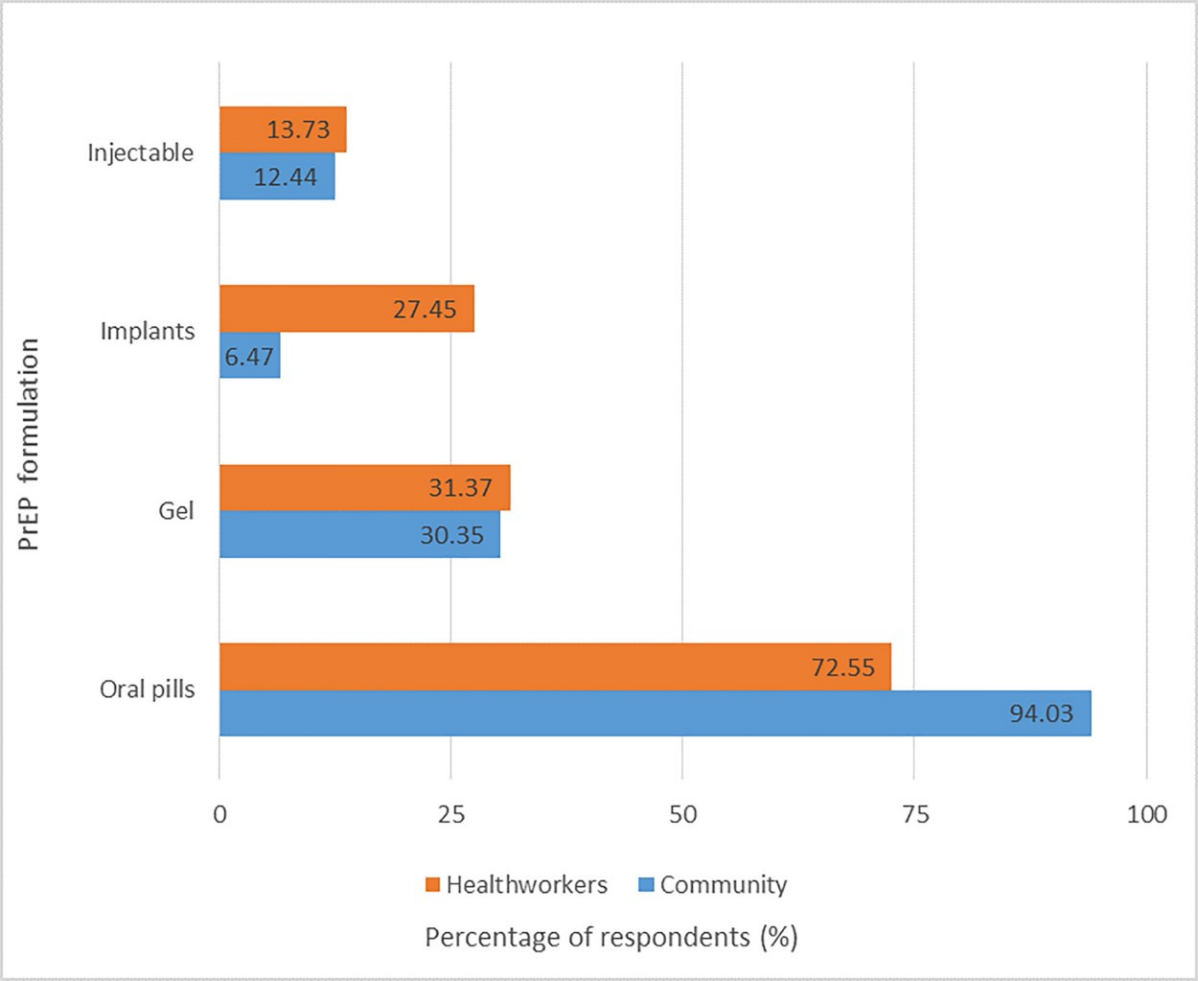
Preferred formulation of PrEP among healthcare workers and community members.

When offered a choice between oral tablets, injectables, gels or implants for PrEP, with the possibility of picking more than one answer, the preferred formulation of PrEP by both health workers and community members was oral pills. However, more health workers (27.5%) preferred implants than community members (6.5%). When asked to choose between taking PrEP daily versus three times per week, more than 80% of the community members were interested in taking PrEP on a daily basis, with less than half (41.8%) interested in taking it less frequently (3 times a week) [Fig 3].

### Perceived personal and health system barriers to PrEP uptake

**Fig 4:**
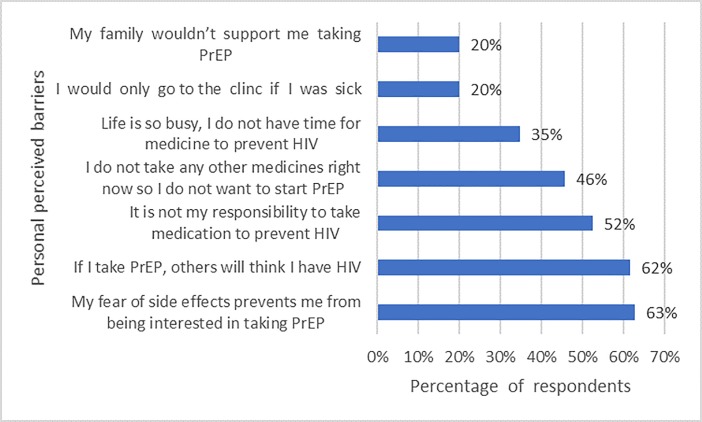
Personal perceived barriers to PrEP among community members.

**Fig 5:**
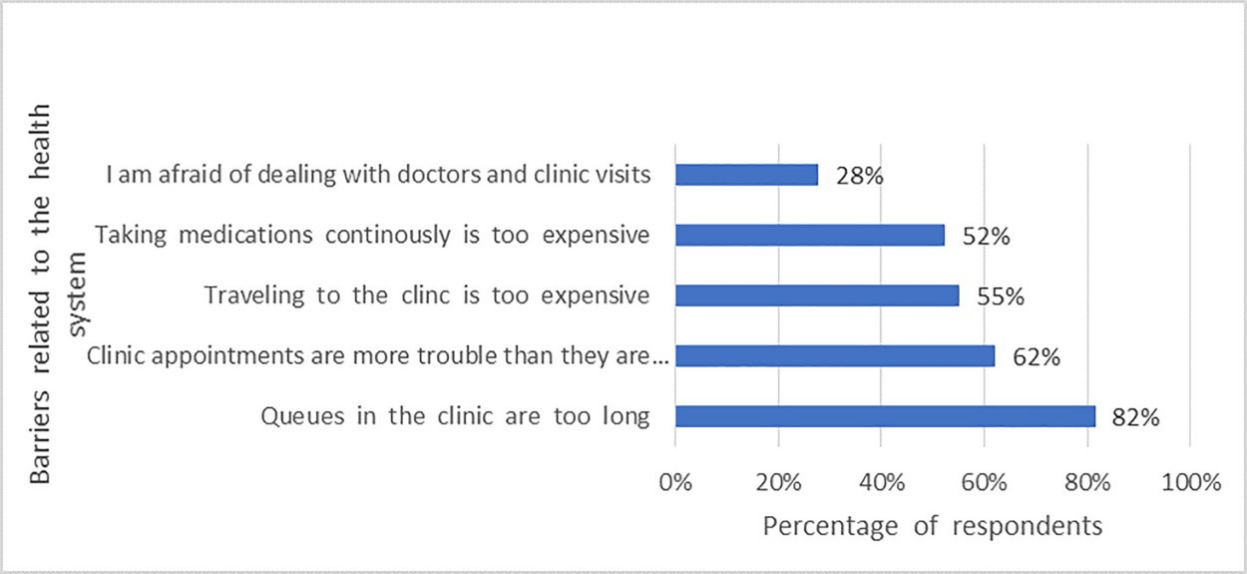
Health system related barriers to PrEP uptake in the community.

Several barriers were reported: 70% of community respondents reported concern for risk of resistance to HIV drugs and 82% of them selected delayed access as the main barrier to PrEP. Other barriers included fear of drug side effects, cost of medication and clinic visits among 63%, 52%, and 63% of respondents from the community respectively (Figs 4 and 5). Stigma was also a major barrier to PrEP among both health care workers and community members. Some of the questions used to assess stigma included, *“If you were taking PrEP*, *others would think less of you”*, *“If you were taking PrEP*, *others would avoid you”*, *“If you were taking PrEP*, *others would think you have HIV”*, *“If you were taking PrEP*, *you would feel comfortable telling others*.*”*

## Discussion

HIV PrEP is a novel public health intervention that holds promise to significantly prevent new HIV infections. However, uptake remains poor in sub-Saharan Africa, as a region, where the majority of these new infections are occurring. Lessons from PrEP studies, where less than expected efficacy occurred, particularly among women who exhibited poor adherence to intervention drugs, are that understanding barriers to adoption of PrEP are essential to informing implementation efforts. Individuals or groups of individuals in each region or country may face unique barriers and challenges to utilizing PrEP.

The study initially assessed the knowledge of individuals about the causation and transmission of HIV infection as these may potentially influence and/or impact their attitudes towards prevention strategies. Our study found, and it was reassuring, that an overwhelming majority of community members correctly identified that HIV infections were caused by a virus, and could be transmitted vertically and by risky sexual behavior. This could be explained by massive nationwide campaigns about HIV primarily run by the Ministry of Health, media coverage on the subject and community-level education at health centers and health posts by community health workers on safe sex practices and other forms of HIV prevention. However, there were also shortfalls in community level knowledge about HIV such as misconceptions surrounding the cause and transmission of HIV in 13.9% and 17.4% of the respondents respectively. These misconceptions fuel unsafe sex practices, promote disease associated stigma and justify the need for more and sustained efforts to educate individuals in the community about HIV.

When asked questions regarding awareness of a drug that could be taken to prevent HIV infection, 86.4% of health care providers (HCPs) and 61.7% of community members agreed to having heard about it but 75% were not able to give correct information regarding the use of PrEP. This disconnect exemplified by high levels of awareness of PrEP as an intervention but with low levels of knowledge about specific details of PrEP shows that there remains a “surface level” understanding about PrEP. It was not surprising that HCPs had higher levels of knowledge than community members as PrEP is now mentioned in local HIV prevention protocols on which HCPs are frequently intimated on [17–19]. A caveat to interpreting the high level of awareness about PrEP may be that both community members and HCPs may confuse PrEP with post exposure prophylaxis.

Health care workers (86.6%) showed similar interest in PrEP as their counterparts from neighboring East-African countries [18], while members of the community from our study were more interested in PrEP compared to data reported from some other African countries (93.5% vs 61% respectively[20]. This variability probably reflects differences in community engagement in and education around HIV prevention tools and strategies.

These high levels of interest in PrEP by both HCWs and community members in Rwanda, once intimated on PrEP, suggest that there is at least an appreciation and appetite for the tool, although this doesn’t always imply that these individuals would prescribe and /or use PrEP. Factors likely to influence the acceptance of PrEP include individuals’ perception of their risk of acquiring HIV infection, but may also represent sociocultural and religious norms about sexuality and sexual behavior. In our study, men from the community were more willing to take PrEP compared to their female counterparts (P = 0.001) as well as those with some level of education, compared to those who hadn’t been to school (P < 0.0001). Women were noted to report much less likely to self-report HIV risk behavior, which may be a true finding or reflect reluctance to disclose HIV risk behavior, which of itself can impede uptake of HIV prevention tools. These findings could also be explained by myths and cultural influences in the community especially among those with low levels of education where a man is perceived as the decision maker in a couples’ sexual life, with women having little or no input especially regarding planning for and decision making within sexual relationships, and having a fear of violence or mistrust from their partners if they utilize HIV prevention tools like PrEP.[21]

Among both groups of respondents, the preferred formulation of PrEP were oral pills. This observation was different from that seen in the Voice-D (MTN-003D) study, where parenteral formulations were the most preferred among participants [19]. Similarly, in a study where HIV negative, sexually active, non-pregnant women in Kenya and South Africa were randomized to receive either tablets, a monthly vaginal ring or intramuscular injection placebo delivery systems for multipurpose prevention of pregnancy and HIV, and subsequently given the choice of any delivery method, the participants expressed a preference for injectables above the other options.[22] The preference of oral pills observed in our study compared to other formulations of PrEP could be explained by lack of sufficient information with regards to the pros and cons of oral pills compared to parenteral formulations, and association of efficacy with HIV treatment formulations that are predominantly oral-based therapies. Also, the preference of oral pills observed in this study was different from that seen in the Voice-D (MTN-003D) study, with parenteral formulations being the most preferred among participants.[19] This could be explained by lack of sufficient information in regards to the pros and cons of oral pills compared to parenteral formulations, given that most of the latter formulations are long acting with a high likelihood of adherence and potentially better outcomes. Certainly, there is an interest on both provider and users for long-acting HIV PrEP formulations.

Barriers to PrEP implementation cited by healthcare workers and community members are quite varied and formidable. Perceived stigma, the time commitment to obtain prevention services from health facilities, medical mistrust and conspiracy theories around HIV are such barriers. Personal-level concerns including lack of family support, reluctance to take a medication daily, fear of being perceived as having HIV and the belief that it is the responsibility of HIV positive partners to utilize measures to prevent transmission are difficult barriers to address. However, other barriers like the fear of side effects (the most frequently cited concern) may be addressed between providers and potential users. For healthcare workers, concerns about the risks of drug resistance, while important, have not been shown to occur as commonly as feared.[23] Cost of the medication, especially in low resource settings, remain a significant impediment to large scale utilization. Similar obstacles are also seen in other parts of the world including countries in sub-Saharan Africa [24–26].

Our study suggests that to optimize PrEP acceptance in Rwanda, community-based efforts have to be made to increase awareness of mode of transmission of HIV which may also lead to decreased stigma associated with PrEP use. One of such strategies could include the use of peer educators which may facilitate acceptance and uptake.[27] For healthcare providers, promoting PrEP uptake and adoption may require implementation of evidence-based models. Furthermore, inclusion of guidance or guidelines that provide a template to inform clinical practice would be essential.[28] Task shifting and utilization of community health workers, as has been shown to be successful for HIV and malaria treatment and prevention efforts,[29, 30] may be helpful to expand PrEP implementation in Rwanda. Cost-effectiveness models may also guide implementation for certain high risk groups which may be impacted by the utilization of generics, where available for PrEP.

Strengths of our study were that we surveyed both potential users and prescribers of PrEP to assess barriers to its adoption. Because of the large number of respondents particularly from a diverse group within the community, the responses likely approximate overall population level beliefs around HIV and PrEP such that it provides valuable information to providers and policymakers on local challenges facing PrEP implementation. Limitations of the study include that the study population might not be representative of the general population given that community members were recruited from a semi-urban area and health care workers were recruited from a tertiary facility whom are the most likely to be aware of current HIV management and prevention approaches. Other study limitations include selection bias as some individuals refused to participate in the survey which may have resulted in non-inclusion of groups such as less educated individuals or others who may have felt uncomfortable responding to surveys. Underreporting/social desirability bias may have impacted self-report on sensitive health issues and behaviors. We did not assess HIV status among survey respondents and this could have influenced their perception about HIV prevention. Finally, we did not assess preference on the dosing frequency of PrEP in our survey and this could have impacted acceptance rates of PrEP.

## Conclusion

Our study shows that while accurate knowledge about HIV and its transmission are high, there is suboptimal knowledge about PrEP. The willingness by community members to take PrEP if indicated is encouraging, however there are multiple individual-level and systemic barriers that were identified and that have to be addressed to optimize its adoption and utilization.

## Supporting information

S1 File PrEP survey tools (English and Kinyarwanda). (DOC)
