# Correction: Perceptions of HIV transmission and pre-exposure prophylaxis among health care workers and community members in Rwanda

**DOI:** 10.1371/journal.pone.0212933

**Published:** 2019-02-22

**Authors:** Immaculate Kambutse, Grace Igiraneza, Sheela Shenoi, Onyema Ogbuagu

Dr. Sheela Shenoi should be included in the author byline instead of the Acknowledgments. She should be listed as the third author, and her affiliation is 2: Yale AIDS Program, Section of Infectious Diseases, Department of Medicine, Yale University, New Haven, Connecticut, United States of America. The contributions of this author are as follows: Conceptualization, Resources.

The correct citation is: Kambutse I, Igiraneza G, Shenoi S, Ogbuagu O (2018) Perceptions of HIV transmission and pre-exposure prophylaxis among health care workers and community members in Rwanda. PLoS ONE 13(11): e0207650. https://doi.org/10.1371/journal.pone.0207650

The following information is missing from the Funding section: SS was supported by National Institute of Allergy and Infectious Diseases (NIAID), grant #K23AI089260. The funder had no role in study design, data collection and analysis, decision to publish, or preparation of the manuscript.
