# Supplementary material for: Perceptions of HIV transmission and pre-exposure prophylaxis among health care workers and community members in Rwanda
Source: PLoS One. 2018 Nov 26;13(11):e0207650. doi: 10.1371/journal.pone.0207650 (PMC6261021; doi:10.1371/journal.pone.0207650)
Supplement: S1 File — (DOC) [file pone.0207650.s001.doc]

## 1. Questionnaire (In English)

**Perceptions of HIV Pre-exposure Prophylaxis, COMMUNITY MEMBERS**

Study Number: ________ Date: ____/____/_____(dd/mm/yyyy)

Site: ⬜ Hospital ⬜ Market ⬜ Taxi Ranks ⬜ College ⬜ Gym ⬜ Other: ______________

*Read prior to asking for Verbal Consent*:

In order to improve our services, we are conducting a survey to understand what community members know about HIV and how they feel about medications to prevent HIV. Would you be willing to answer a brief questionnaire about your knowledge and opinions of HIV and HIV prevention?

Neither your name nor any other information collected will identify you. This questionnaire will not affect your ability to use (or decline) any services today or in the future.

1. Have you provided verbal consent? ⬜ Y ⬜ N

2. Gender: ⬜ Male ⬜ Female

3. Age:________________

4. Marital Status: ⬜ Married ⬜Single ⬜ Partner ⬜Widowed ⬜ Other ______________

5. Nearest Clinic: __________________________________________

6. Ubudehe Category: __________________________________________

7. Health insurance: __________________________________________

Demographics:

| 8. How long does it take to get to your nearest clinic? | | <30min | | <1hr | <2hr | | ≥ 2 hrs | |  | |
| --- | --- | --- | --- | --- | --- | --- | --- | --- | --- | --- |
| 9. How do you get there? | On foot | Taxi | Personal Vehicle | | | | Other | |  | |
| 10. How much does it cost to get to the clinic? Rwf ________ | | | | | | | | |  | |
| 11. Are you employed? | | | | | | Y | | N | | U |
| 12. Did you ever attend school? | | | | | | Y | | N | | U |
| 12a. If yes, How many years of schooling did you complete? | | | | | | □Primary Education □Secondary Education □University | | |  | |

**HIV Knowledge and Perceptions:**

13. How much would you say you know about HIV?

| Nothing | A little | Some | A lot | A great deal |
| --- | --- | --- | --- | --- |

What causes HIV? *(Check all that apply):*

| 14. Germs (Viruses | Y | N | U |
| --- | --- | --- | --- |
| 15. Witchcraft | Y | N | U |
| 16. Drinking alcohol | Y | N | U |
| 17. Smoking cigarettes | Y | N | U |
| 18. Being poor | Y | N | U |
| 19. Punishment from God | Y | N | U |
| 20 Punishment from the ancestors | Y | N | U |

How do you get HIV? *Read the following and tick Y, N, or U for all of the following:*

| 21. Unprotected sex with a person who has HIV | Y | N | U |
| --- | --- | --- | --- |
| 22. Unprotected sex with a person who has HIV but who is on treatment | Y | N | U |
| 23. Sharing food or a drink with someone who has HIV | Y | N | U |
| 24. Breathing air that a person with HIV has coughed into | Y | N | U |
| 25. Sharing clothing with a person who has HIV | Y | N | U |
| 26. Shaking the hand of someone with HIV | Y | N | U |
| 27. From a mosquito that has bitten someone with HIV | Y | N | U |
| 28. From using a toilet after someone who has HIV | Y | N | U |
| 29. Getting pricked by needles or razors that have an HIV infected person’s blood on them | Y | N | U |
| 30. From breast milk | Y | N | U |
| 31. An HIV positive mother can give her child HIV | Y | N | U |

*Ask:* “Do you believe________________?” *Tick Y, N, or U for the following:*

| 32. There is a cure for HIV disease right now | Y | N | U |
| --- | --- | --- | --- |
| 33. HIV is treatable | Y | N | U |
| 34. Once a person starts HIV treatment, he/she must take HIV medications for the rest of his/her life | Y | N | U |
| 35. Condom use can reduce risk of HIV spread | Y | N | U |
| 36. HIV treatment is necessary even if the infected person has no symptoms | Y | N | U |
| 37. People who have AIDS virus have become infected because of their own carelessness^1^ | Y | N | U |

**Perceived Personal HIV Risk**

*Ask*: “Do you agree with any of these statements about HIV?”

| 38. Getting HIV disease can be avoided | Y | N | U |
| --- | --- | --- | --- |
| 39. I worry about getting sick with HIV | Y | N | U |
| 40. HIV is a serious disease | Y | N | U |
| 41. I know someone who has been sick with HIV | Y | N | U |
| 42. I know a family member who has been sick with HIV | Y | N | U |
| 43. I know someone who has died from HIV | Y | N | U |
| 44. Whenever you have sex you are usually the person who suggests using condoms^1^ | Y | N | U |
| 45. If your sex partner does not want to use condoms, there is little you can do about it^1^ | Y | N | U |
| 46. It is easy to reject sexual advances from people who do not want to use condoms^1^ | Y | N | U |
| 47. People who always use condoms won’t get or give the AIDS virus^1^ | Y | N | U |
| 48. In the heat of passion, you have a difficult time using a condom^1^ | Y | N | U |

**Assessment of Preexisting Knowledge of PrEP**

| 49. Have you ever heard of pre-exposure prophylaxis (PrEP)? | Y | N | U |
| --- | --- | --- | --- |

50. How much do you know about HIV pre-exposure prophylaxis (PrEP)?

| Nothing | A little | Some | A lot | A great deal |
| --- | --- | --- | --- | --- |

*Ask:* “Do you agree with any of these statements?”

| 51 | PrEP reduces someone’s risk of getting HIV | Y | N | U |
| --- | --- | --- | --- | --- |
| 52 | PrEP is a medicine that needs to be taken everyday | Y | N | U |
| 53 | If someone takes PrEP, they will not get sick with HIV | Y | N | U |
| 54 | If someone takes PrEP, their partner do(es) not need to use a condom | Y | N | U |

*Read the following:*

There is a medication called pre-exposure prophylaxis (PrEP) that if taken properly, will reduce your risk of getting HIV. If you took it correctly every day, you would be protected from getting sick with HIV for as long as you took the medication. You would have to go to the clinic monthly to get PrEP and have a check-up. The next questions ask how you feel about the idea of taking medication to prevent HIV.

**PrEP stigma:** Read the following, tick Y, N, or U.

| 55 | If you were taking PrEP, others would think less of you | Y | N | U |
| --- | --- | --- | --- | --- |
| 56 | If you were taking PrEP, others would avoid you | Y | N | U |
| 57 | If you were taking PrEP, you would tell a close friend | Y | N | U |
| 58 | If you were taking PrEP, you would think less of yourself | Y | N | U |
| 59 | If you were taking PrEP, others would think you have HIV | Y | N | U |
| 60 | If you were taking PrEP, you would feel comfortable telling others | Y | N | U |

**Self Interest in PrEP and Location:** Read the following, tick Y, N, or U.

| 61 | Are you interested in taking PrEP? | Y | N | U |
| --- | --- | --- | --- | --- |
| 62 | If your doctor recommended PrEP to you to decrease your risk of getting HIV, would you take PrEP? | Y | N | U |
| 63 | Would you take PrEP, if you had to take it 3 times a week in order for it to work? | Y | N | U |
| 64 | Would you take PrEP, if you had to take it once a day, every day in order for it to work? | Y | N | U |
| 65 | Would you take PrEP for as long as needed to prevent getting HIV | Y | N | U |
| 66 | Would you take PrEP even if it did not protect you 100% from getting HIV | Y | N | U |
| 67 | If you were to start PrEP *right now*, would you go to your local health facility to continue treatment every month? | Y | N | U |
| 68 | If you had the choice between going to Health centre or Hospital for PrEP, which would you prefer? | Health Centre | Hospital | U |

**Perceived benefits and barriers:** Do you agree with the following statements?

| 69 | I want to remain HIV negative to take care of my family | Y | N | U |
| --- | --- | --- | --- | --- |
| 70 | I prefer to avoid getting HIV by taking PrEP now | Y | N | U |
| 71 | I want to keep my partner/spouse healthy from HIV | Y | N | U |
| 72 | Taking the PrEP medication can prevent me from getting HIV | Y | N | U |
| 73 | I am not worried about getting HIV, so I do not need medication | Y | N | U |
| 74 | I would set a good example if I took meds to prevent HIV infection | Y | N | U |
| 75 | I am afraid of dealing with doctors and clinic visits | Y | N | U |
| 76 | I do not take any other medicines right now so I do not want to start PrEP | Y | N | U |
| 77 | If I take PrEP, others will think I have HIV | Y | N | U |
| 78 | My fear of side effects prevents me from being interested in taking PrEP | Y | N | U |
| 79 | Life is so busy, I do not have time for medicine to prevent HIV | Y | N | U |
| 80 | Clinic appointments are more trouble than they are worth | Y | N | U |
| 81 | I am treated well when I go to the clinic | Y | N | U |
| 82 | Traveling to clinic is too expensive | Y | N | U |
| 83 | Queues in the clinic are too long | Y | N | U |
| 84 | I would only go to the clinic if I was sick | Y | N | U |
| 85 | My family would support me taking PrEP | Y | N | U |
| 86 | Others would think better of me if I took PrEP | Y | N | U |
| 87 | I would feel protected from HIV if I took PrEP | Y | N | U |
| 88 | I would no longer need to get screened for HIV if I took PrEP | Y | N | U |
| 89 | Taking medications continuously is too expensive | Y | N | U |
| 90 | It is not my responsibility to take medication to prevent HIV | Y | N | U |
| 91 | I am concerned that taking PrEP now will make the HIV drugs not work if I get HIV later | Y | N | U |
| **Skills** | | | | |
| 92 | I know how to take pills | Y | N | U |
| 93 | I remember to take medication daily | Y | N | U |
| 94 | I remember to take pills even while feeling well | Y | N | U |
| 95 | I would avoid missing pills | Y | N | U |
| 96 | I have a place to keep pills | Y | N | U |
| 97 | I can get to the clinic for the refill every month | Y | N | U |
| 98 | I would share information with others about why I’m taking PrEP | Y | N | U |

What Kind of PrEP Formulation would you prefer? (Like= L, Dislike= D, Neutral= N)

| 99 | Oral Pills | L | D | N |
| --- | --- | --- | --- | --- |
| 100 | Injectables | L | D | N |
| 101 | Implants | D | D | N |
| 102 | Gels | L | D | N |
| 103 | Vaginal Inserts/ Rings | L | D | N |

**Would you like to answer the following questions regarding assessment of Personal HIV Risk**

**If yes, Kindly Proceed**

**If No, you are free not to respond**

*Read:*

Sexual activity is one way that a person may get HIV. To better understand the risk of HIV in the community, we would like to know more about the different types of sexual practices people engage in. All of your responses will be kept confidential, but if you feel uncomfortable with any of the following questions, you may decline to answer (D).

Type of Sex Partner^2^:

| 104. Have you had sex in the past month? | Y | N | U | D |
| --- | --- | --- | --- | --- |
| 105. How many partners have you had sex with in the past month? ______________________________ | | | | D |

Beliefs about sexual partner^2^

| 106. Do you believe any of your sexual partners have HIV? | Y | N | U | D |
| --- | --- | --- | --- | --- |
| 107. Do you believe any of your sexual partners have had sex with other people in the past month? | Y | N | U | D |

Sexual Behaviors^2^

108. How often do you have sex with a condom?

| Always | More than half the time | Less than half the time | Never | Declined to Answer |
| --- | --- | --- | --- | --- |

109. Do you drink alcohol before having sex?

| Always | More than half the time | Less than half the time | Never | Declined to Answer |
| --- | --- | --- | --- | --- |

| 110. Has anyone ever paid you for sex? | Y | N | U | D |
| --- | --- | --- | --- | --- |

**Perceptions of HIV Pre-exposure Prophylaxis, HEALTHCARE WORKERS**

Study Number: ________

Date: ____/____/_____(dd/mm/yyyy)

Site: ⬜ Hospital _______________________

⬜ Department______________________(specify clinic)

*Read prior to asking for Verbal Consent*:

In order to improve our services, we are conducting a survey to understand how healthcare workers feel about medications to prevent HIV and what they think their patients know about HIV. Would you be willing to answer a brief questionnaire about your opinions of community member HIV knowledge and HIV prevention? Neither your name nor any other information collected will identify you.

1. Have you provided verbal consent? ⬜ Y ⬜ N

2. Gender: ⬜ Male ⬜ Female

3. Age:________________

4. Marital Status: ⬜ Married ⬜Single ⬜ Partner ⬜Widowed ⬜ Other ______________

**Demographics:**

5. What is your current post?

□ Prof nurse □ Enrolled/staff nurse □ VCT counselor □ Medical officer

□ Other _________________

6. How long have you been a healthcare worker? ___________________________

**HIV Knowledge and Perceptions:**

7. How much would you say your patients know about HIV?

| Nothing | A little | Some | A lot | A great deal |
| --- | --- | --- | --- | --- |

Do you think that your patients believe that HIV is caused by:

*(Check all that apply):*

| 8. Germs | Y | N | U |
| --- | --- | --- | --- |
| 9. Witchcraft | Y | N | U |
| 10. Drinking alcohol | Y | N | U |
| 11. Smoking cigarettes | Y | N | U |
| 12. Being poor | Y | N | U |
| 13. Punishment from God | Y | N | U |
| 14. Punishment from the ancestors | Y | N | U |

Do you think that your patients believe that HIV is caused by:

***Read the following and tick Y, N, or U for all of the following:***

| 15. Unprotected sex with a person who has HIV | Y | N | U |
| --- | --- | --- | --- |
| 16. Unprotected sex with a person who has HIV but who is on treatment | Y | N | U |
| 17. Sharing food or a drink with someone who has HIV | Y | N | U |
| 18. Breathing air that a person with HIV has coughed into | Y | N | U |
| 19. Sharing clothing with a person who has HIV | Y | N | U |
| 20. Shaking the hand of someone with HIV | Y | N | U |
| 21. From a mosquito that has bitten someone with HIV | Y | N | U |
| 22. From using public toilets | Y | N | U |
| 23. Getting pricked by needles or razors that have an HIV infected person’s blood on them | Y | N | U |
| 24. From breast milk | Y | N | U |
| 25. An HIV positive mother can give her child HIV | Y | N | U |

Do you think that your patients believe:

*Tick Y, N, or U for the following*

| 26. There is a cure for HIV disease right now | Y | N | U |
| --- | --- | --- | --- |
| 27. HIV is treatable | Y | N | U |
| 28. Once a person starts HIV treatment, he/she must take HIV medications for the rest of his/her life | Y | N | U |
| 29. Condom use can reduce risk of HIV spread | Y | N | U |
| 30. HIV treatment is necessary even if the infected individual has no symptoms | Y | N | U |
| 31. People who have AIDS virus have become infected because of their own carelessness^1^ | Y | N | U |

***Assessment of Preexisting Knowledge of PrEP***

| 32. Have you ever heard of pre-exposure prophylaxis (PrEP)? | Y | N | U |
| --- | --- | --- | --- |

33. How much would you say you know about HIV PrEP?

| Nothing | A little | Some | A lot | A great deal |
| --- | --- | --- | --- | --- |

34. How often do you talk to your patients about HIV?

| Every visit | More than half the time | Less than half the time | Never | Declined to Answer |
| --- | --- | --- | --- | --- |

*Ask:* “Do you agree with any of these statements?”

| 35. | PrEP reduces someone’s risk of getting HIV | Y | N | U |
| --- | --- | --- | --- | --- |
| 36. | PrEP is a medicine that needs to be taken everyday | Y | N | U |
| 37. | If someone takes PrEP, they will not get sick with HIV | Y | N | U |
| 38. | If someone takes PrEP, their partner do(es) not need to use a condom | Y | N | U |

*Read the following:*

There is a medication called pre-exposure prophylaxis (PrEP) that if taken properly, would reduce an individual’s risk of getting HIV. PrEP is taken daily to prevent HIV infection. If taken correctly every day, their risk of acquiring HIV is decreased.

The next questions ask how you feel about the idea of patients taking medicine to prevent HIV.

**PrEP stigma:** *Ask: Do you agree with the following statements?*

| 39. | If a patient was taking PrEP, others would think less of that patient | Y | N | U |
| --- | --- | --- | --- | --- |
| 40. | If a patient was taking PrEP, others would avoid that patient | Y | N | U |
| 41. | If a patient was taking PrEP, that patient would feel comfortable telling a close friend | Y | N | U |
| 42. | If a patient was taking PrEP, the patient would think less of oneself | Y | N | U |
| 43. | If a patient was taking PrEP, others will think that patient has HIV | Y | N | U |
| 44. | My patients would feel comfortable telling others that they are taking PrEP | Y | N | U |

**Attitudes towards PrEP:** ^Read the following, tick Y, N, or U. 2^

| 45. | I am concerned that PrEP would not protect patients 100% | Y | N | U |
| --- | --- | --- | --- | --- |
| 46. | I am concerned about the potential side effects of PrEP for my patients | Y | N | U |
| 47. | I think patients will be interested in PrEP | Y | N | U |
| 48. | I think patients would take the PrEP medication every day | Y | N | U |
| 49. | I think patients would come to the clinic monthly to get PrEP | Y | N | U |
| 50. | I am concerned that patients will not use condoms if they take PrEP | Y | N | U |
| 51. | I am concerned that patients will have more sexual partners if they take PrEP | Y | N | U |
| 52. | I am concerned that patients using PrEP will no longer get tested for HIV | Y | N | U |
| 53. | I am concerned that taking PrEP continuously would be too expensive for patients | Y | N | U |
| 54. | I am concerned that using PrEP would cause HIV drug resistance | Y | N | U |

**Interest in working with PrEP:** ^Reading the following, tick Y, N, or U 2^

| 55. | I would not recommend PrEP if it is not 100% effective | Y | N | U |
| --- | --- | --- | --- | --- |
| 56. | I have time to counsel patients about ways to decrease HIV risk | Y | N | U |
| 57. | I have time to counsel patients about PrEP | Y | N | U |
| 58. | I believe HIV testing and treating HIV patients are more important than PrEP | Y | N | U |
| 59. | I believe promoting safe sex practices will work better than PrEP to decrease risk of HIV infections | Y | N | U |
| 60. | If available, I would recommend PrEP to my patients right now | Y | N | U |

What Kind of PrEP Formulation would you prefer? (Like= L, Dislike= D, Neutral= N)

| 61 | Oral Pills | L | D | N |
| --- | --- | --- | --- | --- |
| 62 | Injectables | L | D | N |
| 63 | Implants | D | D | N |
| 64 | Gels | L | D | N |
| 65 | Vaginal Inserts/ Rings | L | D | N |

^1^ Center for AIDS Prevention Studies (CAPS). (1995). Voluntary Counseling and Testing Project. AIDSCAP/WHO/CAPS Counseling and Testing Efficacy Study: C & T Baseline Instrument. Retrieved from : [http://caps.ucsf.edu/resources/survey-instruments#11](http://caps.ucsf.edu/resources/survey-instruments#11)

Questions adapted from:

^2^ Puro, V., Palummieri, A., De Carli, G., Piselli, P., & Ippolito, G. (2013). Attitude towards antiretroviral pre-exposure prophylaxis (PrEP) prescription among HIV specialists

. *BMC Infectious Diseases, 13*, 1-8.

**2. Questionnaire in Kinyarwanda**

**Isuzuma ry’Ibitekerezo mu baturajye ku bijyanye n’ikoreshwa ry’imiti yagabanya amahirwe yo kwandura SIDA**

Numero : ________ Itariki: ____/____/_____(dd/mm/yyyy)

Aho uherereye: ⬜ Kwa muganga ⬜ Isoko ⬜ Gare ⬜ Ishuli ⬜ Inzu z’imyitozo Ngororangingo

⬜ Undi mwihariko: ______________

*Soma neza ibi bikurikira*:

Kugira ngo turushe ho kunoza imikorere yacu, turi gukora ubushakashatsi busuzuma ubumenyi ku bijyanye n”indwara ya SIDA mubaturajye, nibitekerezo byabo kubijyanye n’imiti yakoreshwa mu kugabanya amahirwe yo kwandura SIDA. Uremera gusubiza ibibazo bikurikira ku bijyanye n;ubumenyi n’uburyo bwakoreshwa mu kwirinda kwandura agakoko katera SIDA? (umwirondoro wawe ni ibanga, nta ni ngaruka mugihe usubije ibi bibazo)

1. Wemeye gusubiza ibibazo bikurikira? ⬜ Yego ⬜ Oya

2. Igitsina: ⬜ Gabo ⬜ Gore

3. Imyaka Y’amavuko:________________

4. ⬜ Urubatse ⬜Ingaragu ⬜ Inshuti ⬜Umupfakazi ⬜ Undi mwihariko ______________

5. Aho Ivuriro rikwegeyeye riherereye: __________________________________________

6. Icyiciro cy’ubudehe: _________________________________

7. Ubwishingizi bwo kwivuza: _________________________________

irangamimerere ry’ukorerwaho ubushakashatsi:

| 8. Bitwara igihe ki kugera kw’ivuriro rikwegereye? | <iminota 30 | | < Isaha1 | | <Amasaha 2 | | ≥ Amasaha2 |
| --- | --- | --- | --- | --- | --- | --- | --- |
| 9. Uburyo ukoresha kugera Kw’ivuriro rikwegereye | Amaguru | | Taxi | | Imodoka Yawe | | Undi Mwihariko |
| 10. Bitwara Amafaranga angahe kugera kw’ivuriro? Rwf ________ | | | | | | | |
| 11. Ufite akazi? | | Yego | | Oya | | Simbizi | |
| 12. Warize? | | Yego | | Oya | | Simbizi | |
| 12a. Nimba ari Yego, Wize amashuri anagahe? | | □Ayibanze □Ayisumbuye □Kaminuza | | | | | |

**Ubumenyi Ku bijyanye n’indwara ya SIDA:**

13. Ubumwenyi bwawe ku ndwara ya SIDA bungan’iki?

| Ntabwo | Ni buke | Buringaniye | Ni bwinshi | Ni bwinshi cyane |
| --- | --- | --- | --- | --- |

**Ubwandu bwa SIDA buterwa n’iki? (*Y = Yego, O= Oya, S= Simbizi)***

| 14. Udukoko | Y | O | S |
| --- | --- | --- | --- |
| 15. Uburozi | Y | O | S |
| 16. Kunywa Inzoga | Y | O | S |
| 17. Kunywa Itabi | Y | O | S |
| 18. Ubukene | Y | O | S |
| 19. Igihano kuva ku Mana | Y | O | S |
| 20. Igihano kuva kubakurambere | Y | O | S |

SIDA yandurira mur’ ubu buryo? **(*Y = Yego, O= Oya, S= Simbizi)***

| 21. Imibonano mpuzabitsina idakingiye n’umuntu ubana n’ubwandu bwa SIDA | Y | O | S |
| --- | --- | --- | --- |
| 22. Imibonano mpuzabitsina idakingiye n’umuntu ubana n’ubwandu, akaba anafata imiti igabanya ubukana bwa SIDA | Y | O | S |
| 23. Gusangira ibyo kurya/kunywa n’umuntu ubana n’ubwandu bwa SIDA | Y | O | S |
| 24. Guhumeka umwuka umuntu ubana n’ubwandu yakororeye mo | Y | O | S |
| 25. Kwambarana n’umuntu ubana n’ubwandu bwa SIDA | Y | O | S |
| 26. Gukora mu ntoki umuntu ubana n’ubwandu bwa sida | Y | O | S |
| 27. Kuva ku mubu yarumye umuntu ubana n’ubwandu bwa SIDA | Y | O | S |
| 28. Gukoresha ubwiherero bumwe n’umuntu ubana n’ubwandu bwa SIDA | Y | O | S |
| 29. Gusangira ibikoresho bityaye (inzembe, inshinge…) n’umuntu ubana  n’ubwandu bwa SIDA | Y | O | S |
| 30. Mu mashereka | Y | O | S |
| 31. Umubyeyi ubana n’ubwandu bwa SIDA ashobora kwanduza umwana atwite | Y | O | S |

*Uremeranya N’interuro zikurikira?* **(*Y = Yego, O= Oya, S= Simbizi)***

| 32. Kugeza ubu, Nta miti yakiza indwara ya SIDA | Y | O | S |
| --- | --- | --- | --- |
| 33. Hari imiti yakoreshwa n’ababana n’ubwandu bwa SIDA | Y | O | S |
| 34. Mugihe umuntu atangiye gufata imiti igabanya ubukana bwa SIDA, agomba kuyifata ubuzima bwe bwose | Y | O | S |
| 35. Gukoresha agakingirizo, bigabanya amahirwe yo kwandura agakoko gatera SIDA | Y | O | S |
| 36. Ni ngombwa gufata imiti igabanya ubukana bwa SIDA, nubwo umuntu yaba atagaragaza ibimenyetso, mugihe abana n’ubwandu. | Y | O | S |
| 37. Abantu babana n’ubwandu bwa SIDA babitewe n’uburangare | Y | O | S |

**Ibitekerezo ku bijyanye no kwandura SIDA**

*Uremeranya n’interuro zikurikira? ?* **(*Y = Yego, O= Oya, S= Simbizi)***

| 38. SIDA ishobora kwirindwa | Y | O | S |
| --- | --- | --- | --- |
| 39. Kwandura SIDA, bintera ubwoba | Y | O | S |
| 40. SIDA ni indwara ikomeye | Y | O | S |
| 41. Hari umuntu nzi wanduye agakoko gatera SIDA | Y | O | S |
| 42. Hari umuntu mu muryango wanjye ubana n’agakoko gatera SIDA | Y | O | S |
| 43. Hari umuntu nzi wishwe na SIDA | Y | O | S |
| 44. Ni wowe ugira igitekerezo cyo gukoresha agakingirizo buri gihe,mugihe ukoze imibonano mpuza bitsina | Y | O | S |
| 45. Mugihe uwo mukorana imibonano mpuza bitsina yanze gukoresha agakingirizo, ntakindi wakora | Y | O | S |
| 46. Biroroshye guhakanira umuntu, mugihe adashaka gukoresha agakingirizo | Y | O | S |
| 47. Abantu bakoresha agakizirizo nti bakwandura cyangwa ngo banduze virusi itera SIDA | Y | O | S |
| 48. Mugihe wishimiye inshuti yawe, biragoye gufata icyemezo cyo gukoresha agakingirizo | Y | O | S |

**Isuzuma ry’imyitwarire yakwongerera umuntu ibyago byo kwandura virusi itera SIDA**

**isuzuma ry’ubumenyi ku miti yakoreshwa mu kugabanya amahirwe yo kwandura SIDA (PrEP)**

**(*Y = Yego, O= Oya, S= Simbizi)***

| 49. Hari icyo waba warumvise ku bijyane n’imiti yakoreshwa mu kugabanya amahirwe yo kwandura SIDA (PrEP)? | Y | O | S |
| --- | --- | --- | --- |

50. Ubumenyi ufite kuri PrEP (miti yakoreshwa mu kugabanya amahirwe yo kwandura SIDA) bungan’iki?

| Ntabwo | Ni buke cyane | Ni buke | Ni bwinshi | Ni bwinshi Cyane |
| --- | --- | --- | --- | --- |

*Uremeranya n’bi bikurikira?* **(*Y = Yego, O= Oya, S= Simbizi)***

| 51 | PrEP igabanya amahirwe yo kwandura SIDA | Y | N | U |
| --- | --- | --- | --- | --- |
| 52 | PrEP ni umuti ugomba gufatwa buri munsi | Y | N | U |
| 53 | Umuntu aramutse afashe PrEP, ntiyakwandura SIDA | Y | N | U |
| 54 | Umuntu aramutse afashe PrEP, inshuti ye ntigomba gukoresha agakingirizo mu gihe cy’imibonano | Y | N | U |

*Soma ibi bikurikira:*

Hari umuti witwa PrEP wakoreshwa mu kwirinda SIDA, iyo PrEP ifahwe neza kandi buri munsi, igabanya amahirwe yo kwandura agakoko gatera SIDA igihe cyose uyifashe. Umuntu asabwe kujya kw’ivuriro buri kwezi guhabwa imiti hamwe no kwisuzumisha. Ubitekereza ho iki?

**Akato kajyanye na PrEP**(imiti yabanaya amahirwe yo kwandura SIDA)**:** subiza ibibazo bikurikira

**(*Y = Yego, O= Oya, S= Simbizi)***

| 55 | Uramutse ufashe PrEP, abantu bagutakariza icyizere | Y | O | S |
| --- | --- | --- | --- | --- |
| 56 | Uramutse ufashe imiti yabanaya amahirwe yo kwandura SIDA, abantu baguhunga | Y | O | S |
| 57 | Uramutse ufashe PrEP, byakorohera kubibwira inshuti zawe | Y | O | S |
| 58 | Uramutse ufashe PrEP, byatuma witakariza icyiere | Y | O | S |
| 59 | Uramutse ufashe PrEP, abantu batekereza ko ubana n;ubwandu bwa SIDA | Y | O | S |
| 60 | Uramutse ufashe PrEP, byakorohera kubivuga | Y | O | S |

**Ubushake bwo gufata PrEP:** Soma ibi bikurikira

**(*Y = Yego, O= Oya, S= Simbizi)***

| 61 | Hari ubushake bwo kuba wafata PrEP ufite? | Y | O | S |
| --- | --- | --- | --- | --- |
| 62 | Wakwemera gufata PrEP mugihe muganga akugiriye inama yo kuyifata kugira ngo ube wagabanya amahirwe yawe yo kwandura SIDA? | Y | O | S |
| 63 | Wakwemera gufata PrEP inshuro eshatu ku munsi? | Y | O | S |
| 64 | Wakwemera gufata PrEP buri munsi? | Y | O | S |
| 65 | Wakwemera gufata PrEP mugihe cyose uyikeneye, mu rwego rwo kwirinda ubwandu bwa SIDA? | Y | O | S |
| 66 | Wakwemera gufata PrEP, nubwo idakingira umuntu 100% | Y | O | S |
| 67 | Uramutse utangiye gufata PrEP, wajya Kw’ivuriro rikwegereye gufata imiti buri Kwezi ? | Y | O | S |
| 68 | Hagati ya Centre de Sante n’ibitaro, ni hehe wafatira imiti ya PrEP bikworoheye? | **Ikigo nderabuzima** | **Ibitaro** | **S** |

**Inyungu n’inzitizi za PrEP:** Uremeranya n’interuro zikurikira?

**(*Y = Yego, O= Oya, S= Simbizi)***

| 69 | Sinshaka Kwandura SIDA, kugirango nkomeze kwita ku muryango wanjye | Y | O | S |
| --- | --- | --- | --- | --- |
| 70 | Ndifuza kwirinda SIDA, nkoresheje uburyo bwa PrEP | Y | O | S |
| 71 | Ndashaka gukingira umukunzi/umufasha wanjye ubwandu bwa SIDA | Y | O | S |
| 72 | Gufata PrEP, byankingira ubwandu bwa SIDA | Y | O | S |
| 73 | Simfite ubwoba bwa SIDA, Sinkeneye kwirinda nkoresheje PrEP | Y | O | S |
| 74 | Natanga urugero rwiza, mugihe nirinze ubwandu bwa SIDA nkoresheje PrEP | Y | O | S |
| 75 | Mfite ubwoba bw’abaganga | Y | O | S |
| 76 | Sinshaka gufata PrEP kuko ntayindi miti ndi gufata | Y | O | S |
| 77 | Ndamutse mfashe PrEP, abantu batekereza ko nanduye Virusi itera SIDA | Y | O | S |
| 78 | Mfite ubwoba ko nagira ingaruka zikomoka mu gufata PrEP | Y | O | S |
| 79 | Nta mwanya nabona wo gufata PrEP, bitewe n’akazi kenshi ngira | Y | O | S |
| 80 | Umuntu atakaza umwanya muremure kwa muganga | Y | O | S |
| 81 | Mpabwa serivisi nziza iyo ngiye kwa muganaga | Y | O | S |
| 82 | Kujya kwa Muganga birahenze | Y | O | S |
| 83 | Haba imirongo miremire cyane kwa Muganaga | Y | O | S |
| 84 | Njya kwa muganga igihe ndwaye gusa | Y | O | S |
| 85 | Umuryango wanjye wanshyigikira mu gihe niyemeje gufata PrEP | Y | O | S |
| 86 | Abantu bangirira icyizere mu gihe niyemeje gufata PrEP | Y | O | S |
| 87 | Ndamutse mfashe PrEP, nta bwoba bwo kwandura SIDA nagira | Y | O | S |
| 88 | Ndamutse mfashe PrEP, sinakenera kwisuzumisha ubwandu bwa SIDA | Y | O | S |
| 89 | Gufata imiti buri munsi birahenze cyane | Y | O | S |
| 90 | Si inshingano zanjye kunywa imiti mu rwego rwo kurinda ikwirakwizwa ry’ubwandu bwa SIDA | Y | O | S |
| 91 | Mfite impungenge ko ndamutse mfashe PrEP, imiti igabanya ubukana itamvura ndamutse nanduye Virusi itera SIDA | Y | O | S |
| **Ubumenyi** | | | | |
| 92 | Nzi gufata imiti neza | Y | O | S |
| 93 | Nibuka gufata imiti buri gihe | Y | O | S |
| 94 | Nakwirinda gusiba imiti | Y | O | S |
| 95 | Mfite aho mbika neza imiti yanjye | Y | O | S |
| 96 | Najya nitabira kujya kwa muganga buri kwezi gufata imiti | Y | O | S |
| 97 | Najya nsobanurira abantu bitangoye impanvu mfata PrEP | Y | O | S |
| 98 | Nibuka gufata imiti, niyo naba ntarembye | Y | O | S |

Mu buryo bukurikira bwo gukoresha PrEP, ni ubuhe wahitamo? (Yego= Y, Oya= O, Simbizi= S)

| 99 | Ibinini | Y | O | S |
| --- | --- | --- | --- | --- |
| 100 | Inshinge | Y | O | S |
| 101 | agapira gashyirwa munsi y’uruuhu | Y | O | S |
| 102 | Amavuta yo kwisiga (Gel) | Y | O | S |
| 103 | Umuti cg Agapira gakengezwa mu gistina cy’umugore | Y | O | S |

Soma ibi bikurikira:

Bumwe mu buryo butandukanye umuntu yakwandura virusi itera SIDA, harimo n’uburyo bw’imibonano mpuzabitsina. Kugirango turusheho gusobanukirwa ikwirakwiza rya virusi itera SIDA, dukenye gusuzuma ibijyanye n’igikorwa cy’imibonano mpuzabitsina mu baturajye. (Ibisubizo byawe bizagirwa ibanga kandi gusubiza ibi bibazo si agahato)

Niba wemeye gusybiza ibi bibazo bikurikira, wakomeza

Mu gihe utifuza gukomeza, wagarukira hano.

Uwo mukorana imibonano mpuzabitsina^2^: (Y= yego, O= Oya, S= Simbizi, N= Nta gisubizo ntanze)

| 104. Wakoze imibonano mpuzabitsina mu kwezi gushize? | Y | O | S | N |
| --- | --- | --- | --- | --- |
| 105. ni abantu bangahe mwagiranye imibonano mu kwezi gushize? ______________________________ | | | | N |

Ibitekerezo byawe ku bantu mugirana imibonano mpuzabitsina^2^(

Y= yego, O= Oya, S= Simbizi, N= Nta gisubizo ntanze)

| 106. Mu bantu mugirana imibonano, hari uwo utekerezaho ko yaba abana na virusi itera SIDA? | Y | O | S | N |
| --- | --- | --- | --- | --- |
| 107. Mu bantu mugirana imibonano, hari uwo utekereza ko yaba yaragiranye imibonano n’abandi mu kwezi gushize? ? | Y | O | S | N |

108. Ni kangahe ukoresha agakingirizo?

| Buri gihe | Kenshi | Gake | Ntanarimwe | Nta gisubizo ntanze |
| --- | --- | --- | --- | --- |

109. Mbere yo kugira imibonano mpuzabitsina, Nywa Inzoga?

| Buri gihe | Kenshi | Gake | Ntanarimwe | Nta gisubizo ntanze |
| --- | --- | --- | --- | --- |

| 110. Hari ubwo wigize uhabwa amafaranga nyuma y’imibonano mpuzabitsina? | Y | O | S | N |
| --- | --- | --- | --- | --- |

^1^ Center for AIDS Prevention Studies (CAPS). (1995). Voluntary Counseling and Testing Project. AIDSCAP/WHO/CAPS Counseling and Testing Efficacy Study: C & T Baseline Instrument. Retrieved from : [http://caps.ucsf.edu/resources/survey-instruments#11](http://caps.ucsf.edu/resources/survey-instruments#11)

Questions adapted from:

^2^ Corneli, A.L., McKenna, K., Headley, J., Ahmed, K., Odhiambo, J., Skohosana, J…& Agot, K. (2014). A Descriptive Analysis of Perceptions of HIV Risk and Worry about Acquiring HIV among FEM-PrEP participants who seroconverted in Bondo, Kenya, and Pretoria, South Africa. *Journal of the International AIDS Society, 17*, 1-8.

**Ibitekerezo mu batanga serivisi z’ubuvuzi ku bijyanye n’ikoreshwa ry’imiti yagabanya amahirwe yo kwandura SIDA**

Numero : ________

Itariki: ____/____/_____(dd/mm/yyyy)

Aho Uherereye:

⬜ Ibitaro _______________________

⬜ Ishami Ubarizwa mo:______________________

*Soma ibikurikira Mbere Yo gusubiza Ibibazo* :

Kugira ngo turusheho kunoza imikorere yacu, Turi gukora ubushakashatsi , Kugirango dusuzume uko abatanga Serivisi Z’ubuvuzi basobanukiwe ku bijyanye n’imiti ishobora gukoreshwa mu kwirinda virusi itera ubwandu bwa SIDA, Uremera kwuzuza ibibazo bikurikira? (umwirondoro wawe ni ibanga)

1. Wemeye gusubiza ibibazo bikurikira?

⬜ Yego ⬜ Oya

1. Igistina

⬜ Gabo ⬜ Gore

3. Imyaka y’amavuko:________________

4. ⬜ urubatse ⬜Ingaragu ⬜ Ufite Inshuti ⬜ Umupfakazi ⬜ Undi Mwihariko

irangamimerere ry’ukorerwaho ubushakashatsi**:**

5. Aho ukorera?

□ Umuforomo □ Uwimenyereza umwuga W’ubuganaga □ VCT counselor □ Muganga

□Undi Mwihariko _________________

6. Umaze Igihe Kingana iki ukora umurimo w’ubuvuzi? ___________________________

**Ubumenyi n’imitekereze kuri virusi itera SIDA :**

7. Urebye, abarwayi uhura nabo bafite ubumenyi bungan’iki ku bijyanye n;ubwandu bwa

SIDA??

| Ntabwo | Ubumenyi buke cane | Ubumenyi buke | Ubimenyi bwinshi | Ubimenyi bwinshi cane |
| --- | --- | --- | --- | --- |

Ubona Abarwayi batekereza ko ubwandu bwa SIDA buterwa na: *(Yego= Y, Hoya= H, Ntibabizi= N)*

| 8. Udukoko | Y | H | N |
| --- | --- | --- | --- |
| 9. Uburozi | Y | H | N |
| 10. Kunywa Inzoga | Y | H | N |
| 11. Kunywa Itabi | Y | H | N |
| 12. Ubukene | Y | H | N |
| 13. Igihano kuva ku Mana | Y | H | N |
| 14. Igihano kuva Ku bakurambere | Y | H | N |

Ubona Abarwayi batekereza ko ubwandu bwa SIDA buterwa na: *(Yego= Y, Hoya= H, Ntibabizi= N)*

| 15.Imibonano mpuza bitsina idakingiye n’umuntu ubana na virus itera SIDA | Y | H | N |
| --- | --- | --- | --- |
| 16. .Imibonano mpuza bitsina idakingiye n’umuntu ubana n’ubwandu kandi ufata imiti igabanya ubukana bwa SIDA | Y | H | N |
| 17. gusangira ibiryo cg ibyo kunywa n’umuntu ubana n’ubwandu bwa SIDA | Y | H | N |
| 18. Guhumeka Umwuka umwe n’umuntu ubana na Virusi itera SIDA | Y | H | N |
| 19. kwambarana n’umuntu ubana na virusi itera SIDA | Y | H | N |
| 20. Gukoora muntoki umuntu ubana na virusi itera SIDA | Y | H | N |
| 21. Umubu warumye umuntu ubana na virus itera SIDA | Y | H | N |
| 22. Gusangira Umusane Rusange | Y | H | N |
| 23. Ibikoresho bityaye (Inembe, Inshinge) bifite amaraso by’umuntu ubana na Virusi itera SIDA | Y | H | N |
| 24. mu mashereka | Y | H | N |
| 25. ko Umubyeyi ubana na virusi itera sida ya kwanduza Umwana we | Y | H | N |

Ubona Abarwayi uhura nabo batekereza:

*(Yego= Y, Hoya= H, Ntibabizi= N)*

| 26. ko hari umuti ukiza sida | Y | H | N |
| --- | --- | --- | --- |
| 27. Ko SIDA yavurwa n’imiti igabanya ubukana | Y | H | N |
| 28. Ko iyo umuntu atangiye gufata imiti igabanya ubukana bwa SIDA, agomba kuyifata ubuzima bwe bwose? | Y | H | N |
| 29. Ko gukoresha agakingirizo mu gihe cy”imibonano mpuza bitsina byagabanya amahirwe yo kwandura SIDA? | Y | H | N |
| 30. gufata imiti igabanya ubukana ni ngombwa, kabone niyo umuntu ubana na virus itera SIDA yaba adafite ibimenyesto? | Y | H | N |
| 31. abantu banduye agakoko gatera SIDA babitewe n’uburangare | Y | H | N |

**Gusuzuma ubumenyi ku bijyanye n’imiti yagabanya amahirwe yo kwandura SIDA (PrEP)**

| 32. Hari icyo waba warumvise ku bijyanye n’imiti yagabanya amahirwe yo kwandura SIDA | Y | H | N |
| --- | --- | --- | --- |

33. **Ubumenyi bwawe bungan’iki mu bijyanye n’imiti yakoreshwa mu kugabanya amahirwe yo kwandura SIDA (PrEP)**

| Ntabwo | Ni buce cyane | Ni buce | Mfite ubumenyi bwinshi | Mfite ubumenyi bwinshi cane |
| --- | --- | --- | --- | --- |

34. Ni kangahe ugaaniriza abarwayi bawe kubijyanye na Virusi itera SIDA?

| Buri gihe baje kwivuza | Gace cyane | Gace | Ntanarimwe | Nta gisubizo ntanze |
| --- | --- | --- | --- | --- |

*Ask:* “Uremeranya nibi bikurikira?”

| 35. | Hari imiti yakoreshwa mu kugabanya amahirwe yo kwandura SIDA | Y | H | N |
| --- | --- | --- | --- | --- |
| 36. | Umuntu uri gukoresha iyo miti yo kwirinda akeneye kuyifata buri munsi | Y | H | N |
| 37. | Umuntu nti yakwandura virusi itera SIDA mugihe afata imiti yo kwirinda ubwandu | Y | H | N |
| 38. | Si ngombwa ko umutntu akoresha agakingirizo mugihe akoresha imitI yamahirwe yo kwandura virusi itera SIDA | Y | H | N |

*Soma ibi bikurikira*

*Hari imiti yakoreshwa mu kugabanya amahirwe yo kwandura virusi itera SIDA (PrEP), mu gihe ifashwe buri munsi kandi neza.*

Ibitekerezo byawe ku abarwayi bawe bakoresha imiti igabanya amahirwe yo kwandura SIDA (PrEP)

**Akato ka PrEP:** *Uremeranya N’interuro zikurikira?*

| 39. | Kuba umuntu afata imiti yabanaya amahirwe yo kwandura SIDA, byatuma atakarizwa icyizere | Y | H | U |
| --- | --- | --- | --- | --- |
| 40. | Kuba umuntu afata imiti yabanaya amahirwe yo kwandura SIDA, byatuma abandi bamuhunga | Y | H | U |
| 41. | Byakorohera umuntu kubwira incuti ze ko afata imiti yabanaya amahirwe yo kwandura SIDA | Y | H | U |
| 42. | Kuba umuntu afata imiti igabanya amahirwe yo kwandura SIDA,byatuma yitakariza icyiere | Y | H | U |
| 43. | Gufata imiti igabanya amahirwe yo kwandura SIDA, byatuma atekerezwa ho ko abana N’Ubwandu | Y | H | U |
| 44. | Byakorohera abarwayi banjye kwerura ko bafata imiti igabanya amahirwe yo kwandura SIDA | Y | H | U |

**imitekerereze ku bijyanye n’imiti yakoreshwa mu kugabanya amahirwe yo kwandura SIDA (PrEP)**

| 45. | Mfite impungenge ko PrEP itakingira umuntu 100% | Y | H | U |
| --- | --- | --- | --- | --- |
| 46. | Mfite impungenge z’ngaruka zaterwa no gufata PrEP | Y | H | U |
| 47. | Ntekereza ko abarwayi banjye bashimishwa na PrEP | Y | H | U |
| 48. | Ntekereza ko abarwayi banjye bakwitabira gufata PrEP buri munsi. | Y | H | U |
| 49. | Ntekereza ko abarwayi banjye bazajya baza buri kwezi gufata imiti ya PrEP | Y | H | U |
| 50. | Mfite impungenge zuko abarwayi banjye bareka gukoresha agakingirizo mugihe bafata PrEP | Y | H | U |
| 51. | Mfite impungenge zuko abarwayi banjye bashishikarira imibonano mpuza bitsina mugihe bakoresha PrEP. | Y | H | U |
| 52. | Mfite impungenge zuko abantu bareka kwisuzumisha unwandu bwa SIDA mugihe bakoresha PrEP | Y | H | U |
| 53. | Mfite impungenge zuko gukoresha PrEP | Y | H | U |
| 54. | Mfite impungenge zuko gukoresha PrEP byatera resistance y’imiti igabanya ubukana bwa SIDA. | Y | H | U |

**Ubushake bwo gukoresha PrEP:**

| 55. | Sinashishikariza abarwayi gukoresha PrEP mugihe itizewe 100% | Y | H | U |
| --- | --- | --- | --- | --- |
| 56. | Mfite umwanya wo kwigisha abarwayi uburyo bwo kwirinda SIDA | Y | H | U |
| 57. | Mfite umwanya wo kwigisha abarwayi ibijyanye na PrEP | Y | H | U |
| 58. | Mbona gusuzuma SIDA no gutanga imiti ku barwayi babana n’ubwandu ari iby’ingenzi kurusha gukoresha PrEP. | Y | H | U |
| 59. | Mbona gushishikariza abantu gukore imibonano ikingiye ari iby’ingenzi kurusha gukoresha PrEP | Y | H | U |
| 60. | PrEP iramuste ihari, nashishikariza abarwayi guhita bayikoresha | Y | H | U |

Mu buryo bukurikira bwo gukoresha PrEP, ni ubuhe wahitamo? (Yego= Y, Oya= O, Simbizi= S)

| 61 | Ibinini | Y | | O | | S | |
| --- | --- | --- | --- | --- | --- | --- | --- |
| 62 | Inshinge | Y | O | | S | |  |
| 63 | agapira gashyirwa munsi y’uruuhu | Y | O | | S | |  |
| 64 | Amavuta yo kwisiga (Gel) | Y | O | | S | |  |
| 65 | Umuti cg Agapira gakengezwa mu gistina cy’umugore | Y | O | | S | |  |

^1^ Center for AIDS Prevention Studies (CAPS). (1995). Voluntary Counseling and Testing Project. AIDSCAP/WHO/CAPS Counseling and Testing Efficacy Study: C & T Baseline Instrument. Retrieved from : [http://caps.ucsf.edu/resources/survey-instruments#11](http://caps.ucsf.edu/resources/survey-instruments#11)

Questions adapted from:

^2^ Puro, V., Palummieri, A., De Carli, G., Piselli, P., & Ippolito, G. (2013). Attitude towards antiretroviral pre-exposure prophylaxis (PrEP) prescription among HIV specialists

. *BMC Infectious Diseases, 13*, 1-8.
